# Markers of inflammation and influence of nitric oxide on platelet activation in the course of *ulcerative colitis*

**DOI:** 10.18632/oncotarget.19202

**Published:** 2017-07-12

**Authors:** Beata Gawrońska, Joanna Matowicka-Karna, Maciej Kralisz, Halina Kemona

**Affiliations:** ^1^ Department of Clinical Laboratory Diagnostics, Medical University of Bialystok, Białystok, Poland; ^2^ Department of Internal Diseases and Gastroenterology, The Sniadecki Regional Hospital in Bialystok, Białystok, Poland

**Keywords:** ulcerative colitis, platelet activation, nitric oxide, MMP-9, IL-6

## Abstract

Ulcerative colitis is a non-specific inflammatory bowel disease of unknown etiology. We investigated whether severe form of ulcerative colitis may lead to increased number of platelets, changes in platelet parameters and their activation. To address our objectives, we measured concentrations of nitric oxide and markers of inflammation.

We found increased number of low-volume platelets in a group of affected patients. However, their activity was not as high as expected. In addition to that we observed eight times higher concentration of nitric oxide in patients suffering from ulcerative colitis than in healthy individuals. Besides, severe form of the disease manifested itself with increased concentrations of interleukine 6, matrix metalloproteinase-9 and neopterin.

Based on the results we propose that high amounts of nitric oxide inhibit platelet activation in severe form of ulcerative colitis. Moreover, our observations regarding interleukine 6, matrix metalloproteinase-9 and neopterin suggest that they may become useful markers of active form of ulcerative colitis.

## INTRODUCTION

Ulcerative colitis (*colitis ulcerosa*, UC) is a non-specific inflammatory bowel disease of unknown etiology [1–3]. Diffused, non-specific inflammatory process that occurs in the mucosa of the large intestine begins in the rectum and involves the entire large intestine. Interleukin (IL)-6 initiates, enhances and sustains the local inflammatory process. Along with tumor necrosis factor (TNF)-α, interferon (IFN) and bacterial lipopolysaccharide, it activates platelets and stimulates their cytotoxic properties [4]. Platelets can initiate and sustain inflammatory processes through the release of many biologically active substances [5–6]. Released growth factors and chemotactic factors are responsible for the interaction of platelets with phagocytes and fibroblasts. P-selectin is a typical marker of platelet activation. Binding of P-selectin with P-selectin glycoprotein ligand-1 (PSGL-1), which is present on the surface of monocytes and neutrophils, leads to activation of these cells, formation of platelet-leukocyte aggregates and activation of inflammatory and prothrombotic reactions [7–9].

Moreover, induction and maintenance of inflammation is mediated by matrix metalloproteinase 9 (MMP-9) [10–11]. MMP-9 stimulates release of cytokines (TNF-α, transforming growth factor β - TGF-β), activates IL-1β and degrades the basement membrane proteins: collagen IV and lamin. Chronic infection of the colonic mucosa and prolonged activation of MMP-9 expression may lead to the destruction of the basement membrane epithelium. Disintegration of the epithelium resulting from apoptosis of its cells enables migration of lymphocytes and neutrophils to the inflammatory focus. Under such circumstances growth factors are released which then act on surrounding cells [11].

Activated lymphocytes Th1 secrete IFNγ which stimulates production of neopterin by monocytes/macrophages [12]. Neopterin is a non-specific mediator of immune response which stimulates oxidative stress and increases production of oxygen compounds [13].

Nitric oxide (NO) is a factor which inhibits activation and aggregation of platelets and the chemotaxis of neutrophils [14–15]. Pro-inflammatory cytokines: IL-6, IL-17, TNF-α and IFNγ increase production and releasement of NO [16]. Therefore, IL-6 and NO can be considered as indicators of inflammation in the course of UC [17, 18].

The persistent inflammation in patients with ulcerative colitis may be the cause of intravascular platelet activation and changes of their morphological parameters.

The aims of this study were:

## RESULTS

The mean platelet count (PLT) and their morphological parameters in experimental group (UC) and control group (C) and markers of platelets activation (sP-selectin and MPC) are presented in Table 1. In the group of patients with UC, the average of PLT was 304.43 ± 114.46 × 10^3^/μL and was significantly higher compared to the average value obtained in the control group. MPV was significantly lower in the experimental group (UC) than the mean value obtained in the control group.

**Table 1:** The mean platelet count (PLT) and their morphological parameters in experimental group (UC) and control group (C) and markers of platelets activation (as expressed by sP-selectin and MPC)

	Subjects (UC)N=32X ± SD	Control group (C)N=32X ± SD	Statistical significanceP
**Mean platelet count (PLT) (g/L)**	304.43 ± 114.46	247.78 ± 44.74	P < 0.05*
**Mean platelet volume (MPV) (fl)**	8.30 ± 1.08	8.67 ± 0.63	P < 0.05*
**Platelet Distribution Width (PDW) (%)**	52.94 ± 8.87	50.81 ± 3.74	NS
**Large blood platelets (LPLT) (%)**	5.14 ± 3.33	5.35 ± 1.83	NS
**sP-selectin (ng/mL)**	142.40 ± 118.37	130.82 ± 67.13	NS
**Mean platelet component concentration (MPC) (g/dL)**	25.93 ± 2.11	25.70 ± 1.37	NS

N – the number of cases, X –arithmetic mean, SD – standard deviation

*P < 0.05 statistically significant differences, NS – not significant

The coefficient of PDW in patients with ulcerative colitis (UC) was 52.94 ± 8.87% and was slightly higher than the PDW in the control group (C), where the mean value was equal to 50.81 ± 3.74%. This difference was not statistically significant. The average LPLT assessed in both groups did not differ significantly and was to 5.14 ± 3.33% in the experimental group and 5.35 ± 1.83% in the control group.

Slightly higher average concentration of sP-selectin (142.40 ± 118.37 ng/mL) were noted in patients with UC compared to the values in the control group (130.82 ± 67.13 ng/mL). These differences were not statistically significant.

Comparing the values of MPC in patients with UC and in the control group it was noted that the average value in the UC group was 25.93 ± 2.11 g/dL and was almost similar to the value in control group (25.70 ± 1.37 g/dL).

In the group of patients with UC, the average number of red blood cells (RBC) was 4.43 ± 0.38 × 10^6^/μL and was statistically significantly lower (*P*<0.001) compared to the average value obtained in the control group (4.74 ± 0.33 × 10^6^/μL). Mean concentration of hemoglobin in the experimental group (12.88 ± 1.53 g/dL) was statistically significantly lower (*P*<0.0001) than the mean value obtained in the group of healthy controls (14.15 ± 1.03 g/dL).

Results of concentration measurements of nitric oxide (NO), IL-6, WBC, MMP-9 and neopterin are presented in Table 2.

**Table 2:** The concentrations of nitric oxide (NO), IL-6, WBC, MMP-9 and neopterin in both groups (UC and C)

	Subjects (UC)N=32X ± SD	Control group (C)N=32X ± SD	Statistical significanceP
**NO (μmol/L)**	189.67 ± 64.21	22.53 ± 3.14	P < 0.0001*
**IL-6 (pg/ml)**	7.14 ± 3.91	2.45 ± 1.43	P < 0.0001*
**WBC (g/L)**	9.57 ± 2.74	6.58 ± 1.68	P < 0.001*
**MMP-9 (ng/mL)**	13.16 ± 7.27	3.51 ± 0.98	P < 0.001*
**Neopterin (nmol/L)**	2.14 ± 1.67	1.11 ± 0.59	P < 0.001*

N – the number of cases, X –arithmetic mean, SD – standard deviation

*P < 0.05 statistically significant differences

The average concentration of NO in the experimental group was 189.67 ± 64.21 μmol/L, which was eight times higher than the average value obtained in the group of healthy controls (22.53 ± 3.14 μmol/L). The obtained difference is highly statistically significant.

In patients with UC, the mean IL-6 concentration was 7.14 ± 3.91 pg/mL and was significantly higher compared to the average values obtained in the control group (2.45 ± 1.43 pg/mL).

Average number of leukocytes (WBC) in patients with *colitis ulcerosa* equaled 9,57±2,74 g/L and was significantly higher than in healthy persons (6,58±1,68 g/L).

Average concentration of MMP-9 in the test group (UC) equaling 13,16 ± 7,27 ng/mL was significantly higher comparing to the concentration of MMP-9 in the control group (3,51 ± 0,98 ng/mL).

The concentration of neopterin equaled 2,14 ± 1,67 nmol/L in the group of patients diagnosed with *colitis ulcerosa* (UC) and was significantly higher than in a control group (1,11 ± 0,59 nmol/L).

Correlations between particular parameters measured in the group of patients with UC are presented in Table 3.

**Table 3:** Correlation analysis of selected parameters

Pair of variables	Pearson's correlation coefficient (r)
**NO & IL-6**	- 0,1669
**NO & PLT**	0,2414
**NO & MPW**	0,1111
**NO & PDW**	0,0388
**NO & LPLT**	0,1365
**NO & MPC**	- 0,2954

## DISCUSSION

In the course of UC proinflammatory cytokines (IL-6, IL-1, TNF) stimulate the production of acute phase proteins, activate the intrinsic coagulation pathway and induce platelet activation and their cytotoxic properties. Platelets are the key element linking the processes of coagulation, inflammation, and tissue repair. They are involved in inflammatory reactions, and allergic processes through a receptor for IgE (FcεRII and/or FcγRII). In addition, they show the ability to chemotaxis, phagocytosis and diapedesis, so they can phagocytize bacteria, viruses and parasites [4, 19].

In our study, the mean platelet count (PLT) was significantly higher in patients with UC compared to the average value obtained in the control group, which indirectly may indicate stimulation of thrombocytopoiesis. We have shown that MPV was significantly lower in the experimental group compared to the control group, which confirmed the results of studies by other authors [20–22]. On the bases of obtained results MPV indicator can be regarded as a marker of exacerbation of inflammation of colonic mucosa in patients with UC. Güçlü et al. research [23] and our own results confirm that the number of blood platelets increases and the MPV decreases in the active UC. Large platelets “megathrombocytes” (highly reactive cells that are actively involved in the inflammatory response) are gradually consumed to form platelet aggregates and different cell complexes [23–24]. In addition, activated platelets contribute to the formation of microthrombosis in the intestinal microcirculation.

We have shown that the concentration of sP-selectin in patients with UC was higher than in the control group, but these differences were not statistically significant. The increase of sP-selectin concentration indicates platelets stimulation and their involvement in the inflammatory process.

IL-6 may be an indicator of an exacerbation of inflammation in the course of UC in addition to ESR, CRP and WBC [25]. In our studies, it was found that the concentration of IL-6 in patients with UC was almost three times higher than in healthy subjects. Wędrychowicz *et al*. [26] has also shown that the concentration of IL-6 increases in the active UC, and the determination of its concentration in the intestinal biopsy tissue may facilitate the diagnosis of inflammatory bowel diseases [26]. Our results also show that the determination of the concentration of IL-6 may be an useful indicator of an exacerbation of the inflammatory process in the course of UC [17, 26].

Matsuda *et al*. [17] have studied the mRNA expression of IL-6, IL-8, IL-10, TNF-α in the mucosa of patients with active form of UC and in the group of patients in remission phase and in the healthy control group. It has been shown that mRNA levels of all cytokines were higher in patients with active UC than in control group. Additionally it was demonstrated that the mRNA expression of IL-6 and IL-8 in the intestinal mucosa may reflect the activity of UC [17].

Increased levels of IL-6 is accompanied by tightening of ongoing inflammation in the lining of the large intestine, and increased levels of sP-selectin indicates platelet activation and their role in the inflammation process.

Reactive oxygen (ROS) and nitrogen (RNS) species may participate in the development of UC [3]. Adhesion of platelets to neutrophils results in the increase of superoxide anion concentration, which may indicate an interaction of these cells in the inflammatory processes. ROS are products of cellular metabolism (phagocytosis, peroxidation of fatty acids) created during the inflammatory process. Concentrations of antioxidants such as glutathione peroxidase, superoxide dismutase, coenzyme Q10, catalase or metallothionein is reduced in the course of UC [27].

We assessed the concentration of NO, which is an important mediator of acute inflammation and plays a role in repairing damaged tissue. By causing a reduction in the concentration of intracellular Ca^2+^ NO contributes to the relaxation of blood vessels and smooth muscle, the inhibition of the adhesion and aggregation of platelets, the chemotaxis of neutrophils and the signal transduction in the peripheral and central nervous systems [3, 28]. In our study there was achieved a significantly higher concentration of NO in the patients with ulcerative colitis as compared to the control group. High concentrations of IL-6 and NO are associated with the exacerbation of inflammatory process in the large intestine of patients with UC [29]. Other authors [30–31] found that the concentration of NO in blood serum of patients with *colitis ulcerosa* was significantly higher than in patients with remission of UC.

We have shown that the concentration of soluble P-selectin in patients with UC was slightly higher than in the control group, but these differences were not statistically significant. Increased concentration of sP-selectin in the plasma may accompany both inflammatory and cancerous processes [9]. However, it seems that patients with active form of UC lack this sort of response to the inflammation. This fact indicates a presence of potential inhibitors of platelet activation in the experimental group. The most probable candidate is NO which reached on average eight times higher concentration than in the control group. NO is known to be a robust inhibitor of platelet aggregation [32], secretion, adhesion and interaction with fibrinogen [33–35]. NO may inhibit platelet activation via cyclic guanosine monophosphate (cGMP)-dependent and cGMP-independent routes [36–37]. The hypothesis may be verified in the future by treating the UC patients with nitric oxide synthase inhibitors which should lead to the increased activation of platelets.

Our investigations in line with those from other authors [38–39] have proven that concentration of MMP-9 is higher in patients with *colitis ulcerosa* comparing to the control group. MMP-9 belongs to a family of endopeptydases – zinc-dependent proteases. MMP-9 plays a major role in UC pathogenesis and can be a potential therapeutic target [38–40]. It was found that knock-out of *MMP-9* gene in a mouse model limits infection and damage of intestinal mucosa [41–42]. Other authors [43] demonstrated that fecal concentration of MMP-9 in patients with *colitis ulcerosa* was significantly higher than in the control group. It was also correlated with calprotectin level which is a known marker of UC. Results of these investigations allows to conclude that the determination of fecal MMP-9 may become a novel, non-invasive test in differential diagnosis of diarrhea. It may also be used to test the activity of *colitis ulcerosa* and to verify the progress of colonic mucosa recovery [43].

Our investigations showed a statistically significant increase of neopterin concentration in patients with *colitis ulcerosa*. Husain *et al*. [44] demonstrated that neopterin concentration grows proportionally to the degree of *colitis ulcerosa* advancement. Similarly, Nancey *et al*. [45] proved that fecal concentration of neopterin taken from patients with UC was significantly higher than in controls.

Ciećko-Michalska *et al*. [46] found increased concentration of neopterin in blood serum in patients with UC and Crohn disease comparing to the control group. Additionally elevated concentration of neopterin in blood serum is positively correlated with: concentration of TNF-α and CRP, number of leucocytes and blood platelets, and advancement of *colitis ulcerosa*.

Both our findings and those of other authors have shown that the exacerbation of the inflammatory process in the course of UC is accompanied by an increase in platelet count and the reduction of their volume and percentage of LPLT. In addition, the assessment of IL-6, which is a marker of the inflammatory process increases the chances of identification of the exacerbation of inflammation in the mucosa of the intestine. However, the concentration of sP-selectin may reflect its “consumption” in the complex intercellular interactions, including platelet aggregate formation.

## MATERIALS AND METHODS

The study included 32 patients (aged 19-80), 18 men and 14 women, an acute case of ulcerative colitis (UC) who were hospitalized in the Third Department of Internal Medicine and Gastroenterology of the Regional Hospital in Bialystok. Patients’ diagnosis was conducted on the bases on clinical symptoms, laboratory tests and imaging techniques. Following laboratory tests were performed: morphology (WBC, RBC, HGB, HCT, MCV, MCHC, PLT); urine analysis; electrolytes (sodium, potassium, calcium, magnesium); urea; creatinine; bilirubin; glucose; enzymes (alanine aminotransferase, aspartate aminotransferase, amylase, alkaline phosphatase, gamma glutamyltranspeptidase); iron; TIBC; total protein; albumin; prothrombin time; INR; examination of feces. Every patient was examined using blood pressure measurement, electrocardiography and colonoscopy with histopathological examination. In some patients was also performed: ultrasonography of abdominal cavity, RTG of chest, gastroscopy and urease test.

Disease activity was classified according to Truelove and Witts' Criteria (Table 4). Patients were hospitalized for 2 to 56 days (an average 18 days). The majority of patients with ulcerative colitis suffer from secondary anemia, hypertension, giardiosis, gastritis, hemorrhoids, hyperthyroidism, gout, osteoporosis.

**Table 4:** Truelove and Witts' Criteria of severity of ulcerative colitis [47]

Activity	Mild	Moderate	Severe
**Number of bloody stools per day (N)**	< 4	4-6	> 6
**Blood in stool**	rarely	intermediate	often
**Temperature (°C)**	normal	intermediate	> 37,5
**Heart rate (beats per minute)**	normal	intermediate	> 90
**Hemoglobin (g/dL)**	normal	intermediate	< 10,5
**Erythrocyte sedimentation rate (mm/h)**	<20	20-30	> 30

The control group (C) consisted of 32 healthy individuals (aged 18-50 years), including 17 women and 15 men.

Venous blood was collected once prior to the beginning of treatment. 2.7 ml of blood was collected into an anticoagulant - dipotassium edetate (EDTAK_2_) for the determination of morphological parameters of blood platelets and MPC. Concentrations of sP-selectin, NO, IL-6, MMP-9 and neopterin were measured in the serum obtained by 15-minute centrifugation of whole blood venous for at 1000 × g acceleration (obtained samples were collected and stored at <20°C). Samples were frozen from 3 to 6 months.

All patients gave their informed consent to participate in the study, which was approved by the Bioethics Committee of the Medical University of Bialystok, of approved No. RI-002/42/2007.

The assessment of platelet count and their morphological parameters as well as the concentration of MPC were performed on ADVIA 2120 haematology analyzer from Siemens Diagnostics at the Department of Hematology of University Hospital. Concentrations of sP-selectin, NO, IL-6, MMP-9 and neopterin in serum were determined by enzyme immunoassay method using ELISA Kit Human sP-selectin, HS Quantikine ELISA Kit Human IL-6, Total NO/Nitrite/Nitrate Assay, Human MMP-9 R&D Systems and Neopterin ELISA Demeditec Diagnostics GmbH.

The obtained results were statistically analyzed using STATISTICA 10.0 PL. In the experimental and in the control groups an arithmetic mean, minimum and maximum, and standard deviation were calculated. Statistical calculations for the comparison of the experimental and control groups were performed using non-parametric Mann-Whitney U test for two independent samples with distribution different from the normal. The relationships between the two features are described using Pearson's correlation coefficient (r). Results were found to be statistically significant at the significance of *P* <0.05.

## CONCLUSIONS

1. Increment of the number of platelets (PLT) and the reduction of their average volume (MPV) in patients with UC is probably a result of an exacerbation of the inflammatory process.
2. In patients with UC low platelet activation may be a result of the inhibitory action of NO.
3. Increased concentrations of IL-6, MMP-9, NO and neopterin may indicate exacerbation of inflammation during colitis ulcerosa.
